# Social Isolation and Technology usage Among Mexican American Family Caregivers for Alzheimer’s Disease Patients

**DOI:** 10.1093/geroni/igaf122.4197

**Published:** 2025-12-31

**Authors:** Leyi Zhou, Lin Jiang

**Affiliations:** University of California, Berkeley, Berkeley, California, United States; University of Texas Rio Grande Valley, Edinburg, Texas, United States

## Abstract

The number of Latinos affected by Alzheimer’s disease and related dementias (ADRD) is increasing rapidly, with Mexican American family caregivers facing substantial burdens. While information and communication technology (ICT) reduces caregivers’ social isolation, research has predominantly focused on White American populations. This study examines how ICT use affects social isolation and loneliness among Mexican American caregivers, and how cultural and religious factors influence this relationship. In-depth interviews were conducted with 24 Mexican American family caregivers of ADRD patients along the South Texas-Mexico border. Thematic analysis revealed that caregivers experienced social isolation and loneliness that intensified during COVID-19. Cultural factors, particularly familism—the obligation to care for family members—both motivated caregiving but also contributed to isolation when family understanding was limited. ICT use significantly reduced social isolation by maintaining social connections, accessing support groups, and facilitating healthcare communication. ICT effectiveness was influenced by cultural preferences for family-centered communication and varying technology comfort levels. Religion shaped ICT use, with online religious participation serving as both a coping strategy and means of maintaining spiritual community connections. Findings suggest future interventions should prioritize culturally tailored ICT solutions addressing Mexican American caregivers’ specific needs. Understanding the intersection of cultural values, technology use, and social isolation is essential for designing effective support systems that improve quality of life for both caregivers and patients in Latino communities affected by ADRD.

